# Zebrafish as a Useful Tool in the Research of Natural Products With Potential Anxiolytic Effects

**DOI:** 10.3389/fnbeh.2021.795285

**Published:** 2022-01-12

**Authors:** Jonathan Cueto-Escobedo, León Jesús German-Ponciano, Gabriel Guillén-Ruiz, Cesar Soria-Fregozo, Emma Virginia Herrera-Huerta

**Affiliations:** ^1^Departamento de Investigación Clínica y Traslacional, Instituto de Ciencias de la Salud, Universidad Veracruzana, Xalapa, Mexico; ^2^Laboratorio de Neurofarmacología, Instituto de Neuroetología, Universidad Veracruzana, Xalapa, Mexico; ^3^Investigador por México, Consejo Nacional de Ciencia y Tecnología (CONACyT) – Instituto de Neuroetología, Universidad Veracruzana, Xalapa, Mexico; ^4^Laboratorio Ciencias Biomédicas/Área Histología y Psicobiología, Departamento de Ciencias de la Tierra y de la Vida, Centro Universitario de Los Lagos, Universidad de Guadalajara, Lagos de Moreno, Mexico; ^5^Facultad de Ciencias Químicas, Universidad Veracruzana, Orizaba, Mexico

**Keywords:** zebrafish, anxiety, behavioral pharmacology, plant metabolites, translational research

## Abstract

Zebrafish (*Danio rerio*) is a popular and valuable species used in many different biomedical research areas. The complex behavior that fish exhibit in response to different stimuli allows researchers to explore the biological and pharmacological basis of affective and mood disorders. In this sense, anxiety is commonly studied in preclinical research with animal models in rodents. During the last decade, those models have been successfully adapted to zebrafish. Stressful stimuli, such as novel environments, chemical substances, light conditions, and predator images, can trigger defensive behaviors considered indicators of an anxiety-like state. In the first stage, models were adapted and validated with different stressors and anxiolytic drugs with promising results and are now successfully used to generate scientific knowledge. In that sense, zebrafish allows several routes of administration and other methodological advantages to explore the anxiolytic effects of natural products in behavioral tests as novel tank, light-dark chamber, and black/white maze, among others. The present work will review the main findings on preclinical research using adult zebrafish to explore anxiolytics effects of natural products as plant secondary metabolites such as flavonoids, alkaloids and terpenes or standardized extracts of plants, among others. Scientific literature confirms the utility of zebrafish tests to explore anxiety-like states and anxiolytic-like effects of plant secondary metabolites, which represent a useful and ethical tool in the first stages of behavioral.

## Introduction

Anxiety and depression are disorders that cause substantial economic burden (American Psychiatric Association, 2013) and require new treatments with better therapeutic efficacy, fewer adverse effects, and a shorter latency, particularly as antidepressant drugs (Blier, 2003; Nash and Nutt, 2007; Opal et al., 2014). Animal models of anxiety measure behavioral responses in animals, mainly rodents, that are elicited to cope with stressful stimuli and are successfully used to study the biological basis of anxiety and the mechanism of action of anxiolytics and some antidepressants with anxiolytic properties. The increasing use of zebrafish in the last decade is an important advance in preclinical behavioral pharmacology.

The pharmacological screening of natural products to obtain new anxiolytic and antidepressant drugs requires several tests and many animals. In contrast, using small species, such as zebrafish *Danio rerio*, reduces the number of mammals that are used, the maintenance costs, and allows for replacement and refinement in accordance with the 3R's principles of biomedical research in animals (Russell et al., 2005). Zebrafish have unique advantages at the economic and experimental levels that allow medium- and high-throughput pharmacological screening (Giacomotto and Ségalat, 2010; Maximino et al., 2014). However, to exploit these advantages, animal models should be used in ways that meet validity criteria (van der Staay, 2006). The present review discusses the main research in behavioral pharmacology using adult zebrafish models of anxiety as a tool for research of plant secondary metabolites.

Adult zebrafish (*Danio rerio*) has a fusiform body with dark blue longitudinal stripes (Spence et al., 2008). The recommended conditions for captivity are 24–28°C and pH 7–8, with a water hardness of 75–200 mg/L of calcium carbonate (CaCO_3_) and salinity of 0.25–0.6 ppm (Lawrence, 2007). Zebrafish neurotransmitter systems, such as γ-aminobutyric acid (GABA), dopamine (DA), and serotonin (5-hydroxytryptamine [5-HT]), develop during embryogenesis and are functional in larvae (Renier et al., 2007). The brain, vascular system, liver, kidneys, and intestines are fully developed and functional on day 2 after hatching. These findings support the idea of similar aspects of neurochemistry and behavior between mammals and fish that allow the use of zebrafish in behavioral models of anxiety (Chandroo et al., 2004).

## Anxiety Models in Adult Zebrafish

Defensive responses that are exhibited by zebrafish (*Danio rerio*), guppies (*Poecilia reticulata*), and goldfish (*Carasius auratus*) to cope with aversive stimuli have permitted the development of protocols that measure their emotional state based on different behaviors (Maximino et al., 2007). Most behavioral models of anxiety in zebrafish have been adapted from validated rodent models, in which zebrafish are exposed to stressful stimuli, e.g., novel environments, alarm pheromones, illuminated or dark places, and images or dummies of potential natural predators (Cianca et al., 2013), and these modifications are validated with substances that have anxiolytic effects at the clinical level (van der Staay, 2006) allowing the exploration of the potential anxiolytic effects of secondary plant metabolites.

### Light/Dark Test

The light/dark chamber was designed to exploit aversion to brightly illuminated areas and spontaneous exploratory behavior in novel environments in zebrafish as an anxiety index. The device consists of a tank divided into two parts by a central chamber. Half of the device is built with white acrylic, and the other half is built with black anti-reflective acrylic (Maximino et al., 2010a, 2011). Fish usually prefer the dark compartments and avoid illuminated zones. The latency to leave the central chamber and the number of transitions between compartments is also measured (Maximino et al., 2007). This innate preference can be modified by administering clinically effective anxiolytic drugs, such as diazepam, which is interpreted as an anxiolytic-like effect. Nevertheless, illumination preferences vary between studies; in some cases fish preferred white compartments (Cianca et al., 2013) and in others preferred the dark compartments (Maximino et al., 2011). This variation may be the consequence of differences in methodology and tank characteristics; for example, the light intensity: fish spend more time exploring the white compartment when light is around 250 lux, while in lights around 500 lux, fish prefer the dark compartment (Stewart et al., 2011); for that reason, protocol characteristics must be considered in the interpretation of the findings. In the present review, some literature did not specify light intensity, but most of the quoted studies (de Carvalho et al., 2019; Duarte et al., 2019; German-Ponciano et al., 2020; Zenki et al., 2020) illuminated the light/dark chamber thanks with an environmental light bulb of 75 Watts located above the aquarium top (1.8 m), producing a constant average of 975 lux, based on Maximino et al. (2010a,b).

### Black/White Plus Maze

The black/white maze is a variation of the light/dark chamber and elevated plus maze for rodents (Walf and Frye, 2007). The maze is constructed of transparent acrylic with two arms that are configured in a cross shape. The long arm is covered with black polyethylene for the first 10 cm from the center, and the rest is covered with gray polyethylene. The short arms are covered with white polyethylene for the first 10 cm from the center, and the rest is covered with gray polyethylene. During the test (5 min), the number of transitions between arms, and the time spent in the white and central compartments is scored. Like rats, zebrafish exhibit light avoidance that decreases with exposure to anxiolytic drugs (Guo, 2004; Sackerman et al., 2010).

### Novel Tank

The novel tank test is based on the instinctive behavior of zebrafish to seek protection when they are introduced to a novel environment. They prefer to stay on the bottom of the tank until they feel sufficiently secure to explore the entire tank (Egan et al., 2009). During the test (5–6 min) the tank is virtually divided into two equal parts by a horizontal line and each fish is placed individually into the tank. The latency and time of exploration of the upper half of the tank, the number of crossings to the upper half, erratic movements, and time and number of freezing episodes are measured (Bencan et al., 2009). Substances with anxiolytic effects decrease the latency and increase the exploration of the upper parts of the tank (Egan et al., 2009).

### Predator Stimuli

Several protocols have been developed to measure zebrafish responses to predator stimuli. The test device consists of a tank with two LCD computer monitor that is placed along the left and right walls. The monitor allows video presentation of the natural predators of zebrafish, such as gangetic leaf fish (*Nandus nandus*) (Gerlai et al., 2009) and freshwater garfish (*Xenentodon cancila*) (Luca and Gerlai, 2012a). Bird silhouettes can also be displayed by a monitor above the tank (Luca and Gerlai, 2012b). The time in the upper zone of the tank is measured and used as an indicator of emotional state, similar to the novel tank test but in the face of more stressful stimuli, represented by predators (Luca and Gerlai, 2012b). Cianca et al. (2013) developed a robot that mimics the morphology and locomotion of gangetic leaf fish. During the test, the fish-shaped robot is placed in one compartment, and the distance traveled by the zebrafish to escape from the robot and erratic movements are measured.

### Alarm Substances

Zebrafish alarm pheromones are currently unidentified substances that are delivered from wounds in skin that trigger defensive behaviors when detected by other fish, thereby signaling probable proximity to a predator. Behaviors that are triggered by alarm pheromones are measured as anxiety-like behaviors (Speedie and Gerlai, 2008). Alarm substances are obtained by superficially cutting the skin of a decapitated zebrafish. The wounds are washed with distilled water, avoiding contamination with blood. The solution of alarm substances is added to the water after a habituation period (Fontana et al., 2021). The test lasts 7 min, erratic movements, freezing, motor activity, and the distance between fish (when tested in a shoal) are measured (Speedie and Gerlai, 2008). Alarms substances are currently utilized to explore the potential anxiolytic effects of plant extracts as *Coriandrum sativum* (Zenki et al., 2020).

Every model measures several different behaviors under specific conditions, with advantages and disadvantages of each that can influence the choice of a particular model according to the experimental design. Despite these differences, every model triggers the necessary stress to induce behavioral changes that evidence anxiogenic- or anxiolytic-like effects. These changes are equivalent to those observed in rodent models at pharmacological and physiological level, see de Abreu et al. (2020). As would be seen in this review, in adult zebrafish the novel tank and light/dark chamber are the most widely used models of anxiety (Kysil et al., 2017) and have been extensively validated, even as a test battery (Fontana et al., 2021). On the other hand, light-dark transitions are a common anxiety protocol for larvae zebrafish (Peng et al., 2016), which has also been successfully used to screen medicinal plants for potential anxiolytic effects (Maphanga et al., 2020).

## Pharmacological Validation

### Anxiolytic and Antidepressant Drugs

Animal models are always desired to trigger specific responses to drugs that are effective in humans, known as predictive validity (Rodgers et al., 2007). In models of anxiety, clinically effective anxiolytic drugs should decrease anxiety-like behavior in animals. Anxiolytics drugs, such as diazepam, that are administered once by immersion (1.25 and 5 mg/L) reduce the time spent swimming at the bottom of the tank in adult zebrafish, without modifying the velocity of swimming, suggesting an anxiolytic-like effect, and discarding possible motor effects. This effect is not replicated with chlordiazepoxide, in which motor effects appeared at the dose of 5 mg/L. Additionally, some serotonergic drugs that are commonly used as potential antidepressant drugs may produce anxiolytic effects. For example, the 5-HT_1A_ receptor agonist buspirone dose-dependently decreases the time swimming at the bottom of a novel tank when administered by immersion for 3 min (6.25, 25, and 50 mg/L), interpreted as an anxiolytic-like effect. However, this effect is not detected at lower doses (Bencan et al., 2009). Buspirone (5 mg/L by immersion for 60 min) immediately increases swimming near the surface and decreases freezing, but these anxiolytic effects disappeared 3 h after administration, and only motor effects remained (Maaswinkel et al., 2012), suggesting that this test must be performed during the first 2 h after administration. A five-fold higher dose (25 mg/L) produced anxiolytic effects when administered by immersion only for 3 min (Bencan et al., 2009) but produced motor changes that were associated with toxic effects when administered for 60 min (Maaswinkel et al., 2012).

Acute administration of the antidepressants desipramine (25 mg/L) and citalopram (100 mg/L) in adult zebrafish increased swimming near the top of a novel tank. In this case, the hydrophobic drugs were dissolved in dimethylsulfoxide (0.05% DMSO), which has no intrinsic activity in a novel tank. The selective serotonin reuptake inhibitor fluoxetine decreases the latency to swim in the upper part of the tank and increases the time in this compartment after chronic administration in zebrafish (Egan et al., 2009). Finally, in the light/dark chamber, benzodiazepines (0.05 and 1 mg/kg clonazepam, 1.25 mg/kg diazepam, and 0.02 mg/kg chlordiazepoxide) reduce anxiety-like behavior (Maximino et al., 2011). These findings have been confirmed and support the predictive validity of behavioral indicators of anxiety-like states in zebrafish necessary to explore the effect of plant metabolites.

### Effects of Drugs of Abuse on Anxiety

In behavioral pharmacology, drugs of abuse are well-known by their acute anxiolytic effects and the anxiogenic effects triggered during withdrawal. The alkaloid morphine produces anxiolytic-like effects in rats (Anand et al., 2012), which have been replicated in adult zebrafish. Chronic morphine administration, 1.5 mg/L for 2 weeks, exerts anxiolytic effects by reducing freezing and increasing swimming time in the upper zone of a novel tank (Cachat et al., 2011). Additionally, the alkaloid nicotine produces cognitive improvement and anxiolytic effects in mice and humans (Brandon et al., 1999; Xiao et al., 2018). Nicotine administration by immersion (50 mg/L, 5 min) decreases the distance swam and time at the bottom of the tank, interpreted as an anxiolytic-like effect, which is blocked by the nicotinic acetylcholine receptor antagonist mecamylamine, suggesting that the effects are mediated by nicotinic receptors (Levin et al., 2007). Lower doses of nicotine (25 mg/L) had no effects (Sackerman et al., 2010). Lysergic acid diethylamide is a psychedelic drug, the administration of which by immersion (250 μg/L, 15 min) increases swimming in the black chamber in zebrafish, thus demonstrating its anxiolytic activity (Stewart et al., 2011). Another anxiolytic effect is observed in the novel tank with both acute and chronic (7 days) administration of ethanol (0.3%, v/v) for 5 min (Egan et al., 2009).

Fish appear to be as sensitive to addictive drugs as mammals. Cocaine withdrawal produces anxiety in humans (Darland and Dowling, 2001), mice (Craige et al., 2015), and zebrafish. After 5 days of treatment with cocaine chlorhydrate by immersion (1.5 M, 1.5 h), withdrawal produces anxiogenic effects that trigger hyperlocomotion, associated with swim preference at the bottom and walls of the tank. These effects are temporally reduced by 5 μM diazepam (López-Patiño et al., 2008a). Another substance with anxiogenic effects in the light/dark chamber is dipropylcyclopentylxanthine (DCPX; 6 mg/L). DCPX antagonizes adenosine receptors and decreases swimming time in the light compartment (Darland and Dowling, 2001). Caffeine is an alkaloid with anxiogenic effects in rats (Bhattacharya et al., 1997) and humans (Bruce et al., 1992; Smith, 2002). In zebrafish, caffeine administration by immersion (100 mg/L, 15 min) influences behavior in fish, increasing swimming time at the bottom of a novel tank, which has been interpreted as an anxiogenic-like effect (Egan et al., 2009).

## Pharmacological Research on Plant Secondary Metabolites in Zebrafish

Zebrafish has rapidly become a model organism in pharmacology and neuropharmacology. As in rodent, zebrafish have been used to test pharmacological properties of various substances, such as plant extracts and secondary metabolites (Zon and Peterson, 2005). In this sense, zebrafish larvae have been widely used in first for toxicity assessment of crude extracts from plants, specific secondary metabolites or Traditional Chinese medicine (Chahardehi et al., 2020; Zhang et al., 2022) due to the easy exposure to drug and testing that allows high-throughput toxicity screening, the short times of development and low costs. More recently, behavioral protocols with larvae zebrafish using the light-dark transitions have been proposed to explore new drugs from herbal medicine with potential anxiolytic effects (Muniandy, 2018; Maphanga et al., 2020).

Similarly, experimental manipulations have been developed in adult zebrafish to resemble diseases in humans, such as hypercholesterolemia, Parkinson's disease, Alzheimer's disease (Littleton and Hove, 2013), and anxiety (Del Valle-Mojica and Ortíz, 2012). In those studies, zebrafish are advantageous with regard to allowing direct exposure in water, which is useful for elucidating the pharmacological effects of crude plant extracts (Crawford et al., 2008). A great variety of plants, such as *Ceratonia siliqua, Coriandrum sativum, Cymbopogon citratus*, and *Valeriana officinalis*, among others, exert anxiolytic-like effects in zebrafish (Abidar et al., 2020; Mendes et al., 2020; Zenki et al., 2020). Zebrafish are also useful for the determination of active compounds (e.g., flavonoids, polyphenols, terpenes, lignans, and tannins) that may be involved in the therapeutic actions of various plant extracts (Abidar et al., 2020; Lira et al., 2020), mainly through the activation of γ-aminobutyric acid-A (GABA_A_) receptors and interactions with serotonergic and monoaminergic systems (Benneh et al., 2017; Serikuly et al., 2020).

The literature on the effects of plant extract treatments in adult zebrafish evaluated in models of anxiety-like behavior described above are summarized in Table 1. Two important cases are reviewed in more detail below. First, zebrafish are sensitive to pharmacological manipulations of the GABAergic system, which is implicated in the neurobiology of anxiety (Assad et al., 2020). The GABA receptor antagonist pentylenetetrazol produces convulsions. Turmeric oil and a methanolic extract from *Curcuma longa*, together with curcuminoids and sesquiterpenoids (Ar-turmerone, α, β-turmerone, and α-atlantone), have anticonvulsant effects in zebrafish larvae (7 days old). Notably, young larvae are sensitive to GABAergic agents that also regulate the anxiolytic effects of benzodiazepines (Orellana-Paucar et al., 2012). Additionally, a *V. officinalis* extract exerted anxiolytic effects in adult zebrafish, increasing the time spent in the white compartment of the light/dark chamber, similar to the anxiolytic clonazepam. Valerenic acid from *V. officinalis* has the same effects as the extract, suggesting it is the active compound. Zebrafish experiments found that the anxiolytic effect of valerenic acid is blocked by a metabotropic glutamate receptor 1/2 antagonist (Del Valle-Mojica and Ortíz, 2012), suggesting mechanisms beyond simply GABAergic modulation.

**Table 1:** Behavioral effects of plant extracts in adult zebrafish.

**Plant**	**Type of extract**	**Dose**	**Age**	**Model**	**Effect**	**References**
*Valeriana officinalis* roots	Aqueous extract	1 mg/ml	4–6 months	LDC	↑ Time spent in white compartment	Del Valle-Mojica and Ortíz (2012)
*Azadirachta indica* leaves	Hydroalcoholic extract	20 μl/L 40 μl/L	8–9 months 8–9 months	LDC	↑ Entries into white compartment ↑ Freezing	Bernardi et al. (2013)
*Maerua angolensis* stem bark	Aqueous extract	0.1, 0.3, and 1.0 mg/ml	3 months 3 months	LDC NT	↑ Time spent in white compartment ↓ Latency to enter upper section	Benneh et al. (2017)
*Spondias mombin* leaves	Hydroethanolic extract	25 mg/kg, v.o., and 25 mg/L	6 months	LDC	↑ Time spent in white compartment ↓ Latency to enter white compartment ↓ Erratic swimming ↓ Freezing ↓ Thigmotaxis	dos Santos Sampaio et al. (2018)
*Hylocereus polyrhizus*	Lyophilized pulp	0.5 and 1.0 mg/ml	60–90 days	LDC	↑ Time spent in white compartment	Lira et al. (2020)
*Ceratonia siliqua* leaves	Aqueous extract	0.1, 0.3, and 1 mg/L	3–4 months	NT	↑ Time spent at surface ↓ Time spent at bottom	Abidar et al. (2020)
*Coriandrum sativum* leaves	Hydroalcoholic extract	25, 50, and 100 mg/kg, i.p.	4–6 months	LDC	↑ Time spent in white compartment	Zenki et al. (2020)
*Cymbopogon citratus* leaves	Hydroalcoholic extract	1, 3, and 10 g/L	4–6 months	LDC	↑ Time spent in white compartment	Mendes et al. (2020)
*Areca catechu*	Aqueous extract	3, 6, and 12 mg/L	5–7 months	NT	↑ Time spent at surface	Serikuly et al. (2020)

*This table summarizes the research where anxiety models of zebrafish are used to identify the anxiolytic-like effects of plant extracts. L, liter; ml, milliliter, when doses are dissolved in water and administered by immersion. NT, novel tank test; LDC, light/dark chamber; i.p., intraperitoneally; ↑/↓, increase/decrease evaluated variable*.

Second, the neem plant (*Azadirachta indica* A. Juss) is known in ayurvedic medicine for its sedative effects, but it also has toxicity at high doses. The light/dark test in zebrafish confirmed the biological activity of a commercial extract of neem leaves. The immersion of fish in the extract (40 μl/L for 5 min) increased freezing behavior in the white compartment, suggesting an anxiogenic effect that was, however, related to toxic effects (Blaser et al., 2010). In contrast, a lower dose (20 μl/L) produced anxiolytic effects by increasing the number of entries into the white compartment (Bernardi et al., 2013). These effects resemble previous findings in rats, in which failed attempts at exploration in the elevated plus maze were decreased by the administration of an extract of neem (7 mg/kg), which was interpreted as a mild anxiolytic effect (Thaxter et al., 2010). Considering these findings, variations of the effects of neem should be interpreted cautiously by considering specific test conditions and all variables together, rather than separately. Research in zebrafish over the last decade has confirmed the ability to detect anxiolytic-like effects of various plant extracts or secondary metabolites (Muniandy, 2018) and validated as a model for toxicological studies (Cassar et al., 2020). Models in zebrafish have been used as a valuable tool of research of specific groups of substances with potential behavioral effects as flavonoids, which are molecules with potential antioxidant and anxiolytic properties, Table 2 summarize the main findings on anxiety-like behaviors produced with acute and chronic treatment with flavonoids in adult zebrafish.

**Table 2:** Behavioral effects of flavonoids in adult zebrafish.

**Flavonoid**	**Dose**	**Age**	**Model**	**Effect**	**References**
Rutin	50 mg/kg, i.p.	Not specified	LDC	↑ Latency to move in white compartment ↑ Time spent in white compartment	Dubey et al. (2015)
Cinnamaldehyde chalcone	0.1, 0.5, and 1.0 mg/kg, i.p.	60–90 days	LDC	↑ Time spent in white compartment	da Cunha Xavier et al. (2020)
Chalcona PAAMNBA	4.0, 12 y 40 mg/kg, i.p.	60–90 days	LDC	↑ Time spent in white compartment	Ferreira et al. (2020)
Chrysin	1 mg/kg, i.p.	2.5 months	LDC	↑ Time spent in white compartment	German-Ponciano et al. (2020)
Agathisflavone	1, 3 and 5 μg/L	3–4 months	NT	↑ Time spent at surface	Dumitru et al. (2019)
Rhoifolin	1, 3, and 5 μg/L	Not specified	NT	↑ Time spent at surface	Brinza et al. (2020)
Baicalein 5,6-Dimethyl Ether	1, 3, and 5 μg/L	3–4 months	NT	↑ Time spent at surface	Brinza et al. (2021)
Quercetin	1 μg/L,	3 months	NT	↑ Time spent at surface	Zhang et al. (2020)

*Main anxiolytic-like effects produced by flavonoids administered to adult zebrafish and evaluated in different behavioral models. L, liter, when doses are dissolved in water and administered by immersion. NT, novel tank test; LDC, light/dark chamber; OFT, Open Field Test; i.p., intraperitoneally; ↑/↓, increase/decrease evaluated variable*.

Alkaloids are widely distributed secondary metabolites of plants. They are organic compounds that contain one or more nitrogen atoms (in the heterocyclic ring) in the form of a salt (De Luca and St Pierre, 2000). Alkaloids can modify mood and anxiety in zebrafish (Mi et al., 2016; Perviz et al., 2016). The alkaloid Noribogaine in adult zebrafish has been shown to have an anxiolytic effect in the novel tank test, with no effect on locomotion (Kalueff et al., 2017). Ayahuasca (infusion of *Banisteriopsis caapi* stem and *Psychotria viridis* leaves) at low doses (0.1 and 0.5 ml/L for 60 minutes) produce anxiolytic effects in adult zebrafish tested in the novel tank test. While high doses (1 and 3 ml/L for 60 min) produce anxiogenic effects (Savoldi et al., 2017). Serikuly et al. (2020) evaluated the acute and chronic effect of Arecolina at doses of 1 and 10 mg/L in adult zebrafish. Acute treatment produced an anxiolytic effect, with no changes in locomotor activity and body cortisol concentrations. In addition, it increased the concentration of norepinephrine and brain serotonin. On the other hand, it has been shown that l-Scoulerine (l-SLR, 2.5, 5 and 10 mg/L) an alkaloid of tetrahydroprotoberberine (THPBS), reverses in a dose-dependent manner the anxiety-like behaviors induced by methamphetamine in adult zebrafish (Mi et al., 2016) while 10 mg/L of l-SLR had anxiolytic effects *per se*, suggesting that l-SLR may be useful for the treatment of methamphetamine-induced anxiety. Recently, anxiolytic effects of alkaloids present in solanaceous plants have been reported. Nicotine at doses of 0.3 and 30 μM/L and cotinine at a dose of 100 μM/L decreased anxiety-like behaviors in the novel tank test in adult zebrafish (Hawkey et al., 2021). Table 3, summarize the main effects of alkaloids explored in anxiety models using adult zebrafish.

**Table 3:** Behavioral effects of Alkaloid and drugs of abuse in adult zebrafish.

**Alakaloid/drugs**	**Dose**	**Model**	**Effect**	**Reference**
Noribogaine	1, 5 and 10 mg/L.	NT	↑ Time spent and transition to the top half compartment ↓Freezing bouts	Kalueff et al. (2017)
Ayahuasca	0.1, 0.5, 1 and 3 ml/L.	NT	↓ Swimming speed and distance traveled decreased with an increase in ayahuasca ↑ Freezing with 1 and 3 ml/L.	Savoldi et al. (2017)
Arecoline	10 mg/L	NT	Disrupted shoaling, increased social preference, elevated brain norepinephrine and serotonin levels and reduced serotonin turnover	Serikuly et al. (2020)
Tropane (3-(2-methylbutyryloxy)tropan-6,7-diol)	19.5, 38.9 and 116.7 μM/L	NT	↑ Speed while moving, total distance traveled and ↓freezing.	Moreira et al. (2021)
l-Scoulerine Ttetrahydroprotoberberine)	2.5, 5 and 10 mg/L	NT	↑ number of total vertical transitions ↑Time spent at superface	Mi et al. (2016)
Nicotine	1 mg/L	NT, LDC	↑ The time spent in the top and duration of entry in the lit compartment	Duarte et al. (2019)
Caffeine	100 mg/kg	NT, LDC	↑ Thigmotaxis, freezing frequency, and erratic swimming.	de Carvalho et al. (2019)
Nicotine Cotinine	0.3 and 30 μM/L 100 μM/L	NT NT	↑ Distance from the floor (anxiolytic-like effects) ↑ Distance from the floor	Hawkey et al. (2021) Hawkey et al. (2021)

*Main anxiolytic-like effects produced by alkaloids administered to adult zebrafish and evaluated in different behavioral models. L, liter, when doses are dissolved in water and administered by immersion. NT, novel tank test; LDC, light/dark chamber, ↑/↓, increase/decrease evaluated variable*.

Terpenes are volatile and aromatic hydrocarbons, formed by isoprene. Depending on the number of isoprene that form them, they are divided into: (a) monoterpenes, the smallest and most volatile, formed by two isoprene molecules (10C); (b) sesquiterpenes, three molecules of isoprene (15C); (c) diterpenes, four isoprene molecules (20C) and (d) triterpenes, six isoprene molecules (30C); completed by terpenoids, terpenes that contain oxygen in their structure (Hanuš and Hod, 2020; Sommano et al., 2020). Terpenes are the secondary metabolites contained in the essential oils of aromatic plants and responsible for their aroma (Tangpao et al., 2018; Sommano et al., 2020). In addition, terpenes have been shown to have various actions of pharmacological importance, e.g., myrcene the most abundant monoterpene in various cannabis strains has analgesic effect (Jansen et al., 2019), and myrcene, limonene, and β- caryophyllene terpenes found in the essential oil of *Cannabis sativa* (Gulluni et al., 2018) have anti-inflammatory, relaxing, antidepressant and anxiolytic actions in humans (Sharma et al., 2016; Gulluni et al., 2018), rats and mice (Youssef et al., 2019; Song et al., 2021). Similar to observed in models with rodents, in zebrafish it has been described that terpenes such as citral, geraniol or a mixture of both have an anxiolytic-like effects in zebrafish evaluated in the light-dark test, effect reversed by pretreatment with flumazenil, a GABA_A_ receptor antagonist (Mendes et al., 2020) showing that the zebrafish can be an alternative animal model to evaluate the anxiolytic-like effects and the possible mechanism of action of terpenes and its related compounds (Zon and Peterson, 2005). Table 4, summarizes some studies of the anxiolytic potential of terpenes or essential oils with high content of terpenes evaluated in adult zebrafish.

**Table 4:** Behavioral effects of terpenes and essential oils in adult zebrafish.

**Terpene/essential oil**	**Age of evaluation**	**Dose**	**Model**	**Effect**	**Reference**
Citral Geraniol Mixture citral + geraniol	(4–6 months-old)	1, 5 and 10 mg/L 1, 5 and 10 mg/L 1 mg/L + 1 mg/L	LDC	↑ Latency to move in white compartment ↑ Time spent in white compartment ↑ Time spent in white compartment ↑ Time spent in white compartment	Mendes et al. (2020)
*Thymus vulgaris* L., essential oil (97% of the oil are terpenes) thymol (42.10%), p-cymene (19.20%) and β-caryophyllene (6.40%).	(3–4 months-old)	25, 150, and 300 μL/L	NT	↑ Time spent at surface	Capatina et al. (2020a)
*Rosmarinus officinalis* L., essential oil (> 70% of the oil are terpenes) like eucalyptol (26.02%), α-pinene (19.89%), camphor (16.71%), camphene (8.67%), β-myrcene (3.97%), β-caryophyllene (3.11%), borneol (2.50%), and limonene (2.16%)	(3–4 months-old)	25, 150, and 300 μL/L	NT	↑ Time spent at surface	Capatina et al. (2020b)
Limonene Linalool β-myrcene Limonene β-myrcene	Adult zebrafish	0.25%, 0.5% and 0.75% 0.0001%, 0.001% and 0.00125% 0.001%, 0.01% and 0.1% (0.39%) (0.0083%)	NO	↑ Time spent in the center and transition zones No effect ↑ Time spent in the center and transition zones No effect No effect	Szaszkiewicz et al. (2021)

*Main anxiolytic-like effects produced by terpenes or essential oils with high content of terpenes administered to adult zebrafish by immersion and evaluated in different behavioral models. NT, novel tank test; LDC, light/dark chamber; NO, Novel object approach test; ↑/↓, increase/decrease evaluated variable*.

## Neurobiology of Anxiolytic Effects in Zebrafish

The presence of completely functional benzodiazepine receptors in fish was confirmed 25 years ago in *Pimephales promelas* (Rehnberg et al., 1989). The distribution of GABAergic neurons in zebrafish has been described immunohistochemically and by Nissl staining in the olfactory bulb, telencephalon, tectum stratum, and hypothalamus (Kim et al., 2004). The *in vitro* extracellular activity of neurons showed higher activity when GABA_A_ receptors were pharmacologically antagonized with bicuculline methiodide, suggesting a completely functional inhibitory role of GABAergic neurons in zebrafish (Kim et al., 2004). Similarly, zebrafish are sensitive to drugs that act on the GABAergic system, such as pentylenetetrazol, which induce convulsions. In contrast, pretreatment with diazepam attenuates convulsions (Mussulini et al., 2013).

Additionally, the serotonergic system has also been implicated in modulating the therapeutic effects of anxiolytic drugs in zebrafish (Maximino, 2012). Zebrafish possess a fully developed serotonergic system (Stewart et al., 2010) involved in regulating different zebrafish behaviors, such as motor function, aggression, and anxiety-like responses, among others (Maximino and Herculano, 2010; Maximino et al., 2013). The zebrafish serotonergic system has anatomical and genetic similarities to mammals but also important differences (Connors et al., 2014). For example, like mammals, serotonergic neurons in zebrafish are located in raphe nuclei (Jacobs and Azmitia, 1992; Lillesaar et al., 2007, 2009; Lillesaar, 2011). The anxiolytic effect of SSRIs has been evaluated in zebrafish. Studies have shown that chronic SSRIs treatment exerts anxiolytic effects in zebrafish, similar to rodents and humans (Egan et al., 2009; Stewart et al., 2010). However, the anxiolytic effect of acute SSRIs treatment has only been observed in humans and rodents (Bagdy et al., 2001; Drapier et al., 2007), whereas acute fluoxetine administration has no effects in zebrafish (Stewart et al., 2010). The pharmacological activation of serotonin receptors has generally been suggested to promote a reduction of anxiety-like behavior in zebrafish (Sackerman et al., 2010; Gebauer et al., 2011; Maaswinkel et al., 2012; Connors et al., 2014).

Substances of vegetal origin also produce anxiolytic-like effects in zebrafish through serotonergic mechanisms. Benneh et al. (2017) reported that an aqueous extract of *Maerua angonelis* (0.1, 0.3, and 1.0 mg/ml by immersion) increased the time spent in the white compartment in the light/dark chamber. It was associated with actions on 5-HT_1−3_ receptors. Pretreatment with methysergide (a 5-HT_2B/2C_ receptor antagonist), pizotifen (5-HT_2A/2C_ receptor antagonist), and granisetron (a 5-HT_3A/3B_ receptor antagonist) blocked the anxiolytic-like effect of *Maerua angonelis*. Likewise, synthetic chaconne (a main precursor in flavonoid biosynthesis) 4'-[(2E)−3-(3-nitrophenyl)-1-(phenyl) prop-2-en-1-one] acetamide (PAAMNBA**;** 4, 12, or 40 mg/kg, i.p.) increased the time spent in the white compartment in the light/dark chamber. Cyproheptadine, a 5-HT_2A_ receptor antagonist, and pizotifen, a 5-HT_1_ and 5-HT_2A/2C_ receptor antagonist attenuated the anxiolytic-like effect of PAAMNBA (Ferreira et al., 2020). This evidence supports an equivalent neuropharmacological substrate between zebrafish and rodents in regulating anxiety-like behaviors, suggesting that zebrafish is valuable as rodents in behavioral pharmacology investigations of anxiety.

## Final Comments

Pharmacological research on new drugs usually begins at the computational or molecular level in *in sillico or in vitro* experiments that measure or manipulate the activity of molecular targets that are involved in pathology or the mechanism of action of known therapeutic agents. The next stage evaluates toxic and therapeutic effects in animals to confirm biological activity and improve treatment safety as a prelude to studies in humans. The automatization of *in vitro* experiments allows the use of high-throughput techniques to test many substances quickly. However, the results often differ when tested in whole animals because of various pharmacokinetic effects. Additionally, *in vitro* experiments only allow testing effects on specific known molecular targets that are previously characterized, without the possibility of discovering new molecular targets. The protocols described herein are being adapted to zebrafish larvae that can be tested on enzyme-linked immunosorbent assay plates to allow high-throughput screens using whole animals, thereby overcoming pharmacokinetic differences. Notably, high-throughput screening in whole zebrafish will allow the discovery of new molecular targets and effects of plant secondary metabolites.

Finally, to our knowledge, there are a lack of studies of the role of sex in the expression of anxiety- and depression-like behavior in zebrafish. This is an important concern because of sexual dimorphism of the development of anxiety in men and women (Altemus et al., 2014; McHenry et al., 2014), which has also been confirmed in rodent models (Carrier et al., 2015). For example, there are gender differences in anxiety that is induced by cocaine withdrawal in zebrafish. Such withdrawal-induced anxiety is expressed as hyperactivity and stereotypy that develops rapidly and transiently in female zebrafish, whereas it is slow and intense in male zebrafish. However, the effects of withdrawal on the dopaminergic system and the behavioral anxiogenic effects of N-methyl-β-carboline-3-carboxamide (an anxiogenic benzodiazepine receptor inverse agonist) were similar between males and females (López-Patiño et al., 2008b). Similarly, sexual dimorphism of the effects of alcohol exposure was observed in wildtype and long-fin striped zebrafish. Female zebrafish were more sensitive to the effects of ethanol than males, measured by the distance between each fish and its nearest neighbor in a group of eight fish. Chronic ethanol exposure increased the cluster distance more markedly in males than in females (Dlugos et al., 2011). Although the test that was used is not validated as a model of anxiety, the clustering distance in zebrafish has been proposed to measure anxiety- and stress-related behaviors (Maaswinkel et al., 2012, 2013). Exploring the effects of sex on behavioral indicators of anxiety in zebrafish is important and encouraged by US and Canadian health agencies that seek the incorporation of sex as a biological variable affecting other effects in preclinical and clinical research (Clayton, 2018). In other species, such as rodents, the anxiolytic effects of some drugs and plant extracts vary over the course of the ovarian cycle. For example, extracts of *Montanoa frutescens* and *Montanoa grandiflora* and the anxiolytic drug diazepam produced anxiolytic-like effects, particularly during metestrus-diestrus, in rats, but no significant effects of these extracts or diazepam were detected in the elevated plus maze during proestrus-estrus (Rodríguez-Landa et al., 2014); similar effects occurs with isolated secondary metabolites as caffeine (Guillén-Ruiz et al., 2021). To our knowledge few works have explores sex dimorphism in the anxiolytic effects of secondary plant metabolites zebrafish (dos Santos et al., 2021) a feature that remain to be extensively explored.

## Conclusion

Behavioral pharmacology research in zebrafish will continue to expand because of the unique advantages of this model species. In adult zebrafish, the novel tank and light/dark chamber have become commonly used among the wide variety of models. Models of anxiety in adult zebrafish have become useful for identifying potential secondary plant metabolites with potential anxiolytic effects.

## Author Contributions

JC-E and LG-P structured the review. All authors (JC-E, LG-P, GG-R, CS-F, and EH-H) participated in reviewing literature, writing and editing the text and agreed to the final version.

## Funding

JC-E received a grant from Federal Government Program PRODEP (UV-PTC-900) and SNI from CONACYT (CVU 171055). LG-P received a fellowship from Consejo Nacional de Ciencia y Tecnología (CONACyT) for postgraduate studies in neuroethology (Reg. 297560). GG-R received a grant from Investigadores por México-CONACYT project #1840 and SNI from CONACYT (CVU: 285449).

## Conflict of Interest

The authors declare that the research was conducted in the absence of any commercial or financial relationships that could be construed as a potential conflict of interest.

## Publisher's Note

All claims expressed in this article are solely those of the authors and do not necessarily represent those of their affiliated organizations, or those of the publisher, the editors and the reviewers. Any product that may be evaluated in this article, or claim that may be made by its manufacturer, is not guaranteed or endorsed by the publisher.
